# Eosinophil Count and Neutrophil-Lymphocyte Count Ratio as Prognostic Markers in Patients with Bacteremia: A Retrospective Cohort Study

**DOI:** 10.1371/journal.pone.0042860

**Published:** 2012-08-09

**Authors:** Roser Terradas, Santiago Grau, Jordi Blanch, Marta Riu, Pere Saballs, Xavier Castells, Juan Pablo Horcajada, Hernando Knobel

**Affiliations:** 1 Department of Epidemiology and Health Services Evaluation, Hospital del Mar, Barcelona, España; 2 PhD programme in Medicine, Universitat Autònoma de Barcelona, Barcelona, España; 3 Department of Pharmacy, Hospital del Mar, Barcelona, España; 4 Universitat Autònoma de Barcelona, Barcelona, España; 5 CIBER de Epidemiología y Salud Pública (CIBERESP), Barcelona, España; 6 Department of Internal Medicine and Infectious Diseases, Hospital del Mar, Barcelona, España; Public Health Agency of Barcelona, Spain

## Abstract

**Introduction:**

There is scarce evidence on the use of eosinophil count as a marker of outcome in patients with infection. The aim of this study was to evaluate whether changes in eosinophil count, as well as the neutrophil-lymphocyte count ratio (NLCR), could be used as clinical markers of outcome in patients with bacteremia.

**Methods:**

We performed a retrospective study of patients with a first episode of community-acquired or healthcare-related bacteremia during hospital admission between 2004 and 2009. A total of 2,311 patients were included. Cox regression was used to analyze the behaviour of eosinophil count and the NLCR in survivors and non-survivors.

**Results:**

In the adjusted analysis, the main independent risk factor for mortality was persistence of an eosinophil count below 0.0454·10^3^/uL (HR = 4.20; 95% CI 2.66–6.62). An NLCR value >7 was also an independent risk factor but was of lesser importance. The mean eosinophil count in survivors showed a tendency to increase rapidly and to achieve normal values between the second and third day. In these patients, the NLCR was <7 between the second and third day.

**Conclusion:**

Both sustained eosinopenia and persistence of an NLCR >7 were independent markers of mortality in patients with bacteremia.

## Introduction

Total leukocyte and neutrophil count has historically been used as a marker of infection. An association has been found between the presence of infection and monocyte and lymphocyte counts, as well as specific associations between these two counts [1], [2]. In 1922, Simon [3] coined the term “septic factor” to describe an association between neutrophilia and eosinopenia, and considered this factor a useful sign to guide diagnosis of pyogenic infection. This author also suggested that an increase in eosinophils could indicate that recovery had begun. Several studies have used eosinophil counts, specifically eosinopenia, as a marker of infection [4]–[8] and as an indicator of bacteremia [9]–[11], although the results are controversial.

In 2003 Gil et al. [6] showed that eosinophil count was a marker of infection, demonstrating that a leukocyte count of above 10,000/mm^3^ and an eosinophil count of below 40/mm^3^ were strongly related to the presence of bacterial infections.

Subsequently, Abidi et al. [7] evaluated eosinophil count as an indicator of sepsis and suggested that eosinopenia could be useful as a marker of infection in daily clinical practice.

Several biomarkers, such as C-reactive protein and procalcitonin, have been used to indicate bacterial infection. These biomarkers could also provide prognostic information in distinct infectious processes and in patients with sepsis [12]–[15]. These biomarkers have limited sensitivity and specificity but the greatest limitation of procalcitonin is probably its high cost, placing it practically out of the reach of developing countries.

A few studies have analyzed eosinophil count as a prognostic marker of outcome in patients with infection [16], [17], but its utility as a marker of outcome in patients with bacteremia is unknown.

## Materials and Methods

### Aim

To evaluate whether changes in eosinophil count, as well as the neutrophil-lymphocyte count ratio (NLCR), could be used as clinical markers of outcome in patients with bacteremia.

### Design

A retrospective cohort study in patients with a first episode of bacteremia either during admission or when presenting to the emergency department was carried out.

This study was approved by an independent ethics committee. No additional informed consent was required.

### Participants

Patients admitted to the *Hospital Universitario del Mar* in Barcelona, Spain, with a first episode of community-acquired or healthcare-related bacteremia between 2004 and 2009.

The hospital has a bacteremia surveillance team that prospectively follows up all patients with an episode of bacteremia. Bacteremia or fungemia was defined as the presence of bacteria or fungi in blood identified through blood culture (henceforth referred to as bacteremia to reflect the two etiologies). Healthcare-associated bacteremia was defined as the presence of an infectious agent documented 3 days after the patient’s admission to the hospital with no evidence that the infection was present or incubating at the time of admission [18], [19]. Blood cultures considered contaminated were excluded from the study. A culture was considered contaminated if a common skin contaminant i.e., coagulase-negative *Staphylococcus*, *Bacillus* spp., *Propionibacterium acnes*, or *Corynebacterium* spp was isolated in only one blood culture sample from the same patient. The criteria used for the sources of bacteremia were the CDC/NHSN surveillance definition [18]. When no focus of infection causing the bacteremia was identified, the source was considered unknown. Blood samples were collected following the hospital’s pre-established protocols, using a sterile technique and peripheral veins. All data were drawn from clinical practice.

Patients aged less than 18 years old, as well as those with haematological cancer, HIV infection, or an eosinophil count above the upper limit of normality caused by parasitic diseases were excluded from the cohort. Patients with a second episode of bacteremia in a single admission were also excluded because recurrent episodes of bacteremia have been independently associated with increased mortality [20]. Patients with a single laboratory determination were excluded from the analysis of distinct leukocyte counts. However, the data from these patients were used to determine the value of the baseline counts in the bacteremia episode.

### Variables

The dependent variable was crude mortality 15 days after documented bacteremia. The main explanatory variables were eosinophil count and the NLCR.

The remaining explanatory variables consisted of the patients’ demographic data (age, sex), date of blood culture, source of the infection causing the bacteremia, the microorganisms isolated, type of admission (elective or emergency), admission date, reason for admission (medical or surgical), corticosteroid use, and vasopressor use. To evaluate comorbidities, the Charlson index was used [21].

When more than one laboratory test was carried out on the same day, only the first was included. To perform the analyses, the microorganisms identified were divided into distinct groups: monomicrobial bacteremia, classified as *Escherichia coli, Klebsiella spp, Pseudomonas aeruginosa,* anaerobic microorganism, other Gram-negative microorganisms, *Staphylococcus aureus, Streptococcus pneumoniae*, other Gram-positive microorganisms, fungus, and polymicrobial bacteremia.

Normal values in the leukocyte series were as follows: leukocytes 4–11.0 10^3^/uL, neutrophils 2.5–8.2 10^3^/uL, lymphocytes 1.5–5.0 10^3^/uL, eosinophils 0.05–0.5 10^3^/uL, monocytes 0.2–1.0 10^3^/uL, basophils 0–1.23.4 10^3^/uL. The haematology analyzer used in the laboratory was a Sysmex XT-1800i. (Sysmex Asia Pacific Pte Ltd and Sysmex Corporation of Japan).

The data collected by chart analysis consisted of blood cell counts and the Charlson comorbidity index. All the remaining variables analyzed were obtained when visiting the patients.

### Statistical Methods

The primary outcome was crude mortality at 15 days after documented bacteremia. The categorical variables were expressed as counts and crude mortality rates. The continuous variables were expressed as the mean, standard deviation (SD), median and the interquartile range (IQR). Categorical variables were compared using the chi-squared test and continuous variables were compared using the Mann-Whitney U-test.

The eosinophil count was classified into three categories defined by distribution tertiles. Another categorization was studied, but tertiles were the easiest to interpret and had the best fit. Eosinophil count tertiles were defined as below the normal range (0·10^3^/uL to 0.0453·10^3^/uL), low but within the normal range (0.0454·10^3^/uL to 0.1510·10^3^/uL) and high but within or above the normal range (0.1511·10^3^/uL to a maximum of 1.4415·10^3^/uL). In addition, the NLCR was classified into two categories using the median. As for eosinophil count, we studied another categorization and the median showed the best fit. The NLCR were labelled as high ratio (NLCR >7) and normal ratio (NLCR ≤7).

The Kaplan-Meier method was used to estimate the cumulative probability of patient survival according to eosinophil count [22]. As eosinophil count is a time-dependent variable, the Kaplan-Meier curves were estimated using the Nelson-Aalen estimator to correct for time-dependent bias [23]. To compare the Kaplan-Meier curves, we used the log rank test, with a univariate Cox regression model. To use this method, the eosinophil counts for each patient in all observed days were interpolated linearly to obtain a hypothetical curve between blood measurements. For each day, this curve was compared between survivors and non-survivors with the Mann-Whitney U-test.

A Cox regression with proportional hazard was performed to evaluate differences in survival among patients with different levels of eosinophil counts adjusted by the covariables. Because of the time-dependent nature of the eosinophil counts and NLCR, a Cox model with time-dependent covariates was applied [22], [24]. Differences in survival were evaluated with unadjusted and adjusted hazard ratios (HR) and their 95% confidence intervals (95%CI). The hypothesis of proportional hazard was tested through log-log survival curves. In addition, to determine whether eosinophil count behaves differently in each strata, we performed an analysis stratified by vasopressor use.

A logistic model was performed to establish the prognostic value of the baseline measurement of eosinophil and NLCR in crude mortality at 3 days. The area under the receiver operating characteristic (ROC) curve determined the discriminatory power of the baseline measurement and its predictive value.

The statistical analysis was performed using the R program, version 2.13.0 [24]. All p-values were bilateral, and p-values <0.05 were considered statistically significant.

## Results

**Figure 1:**
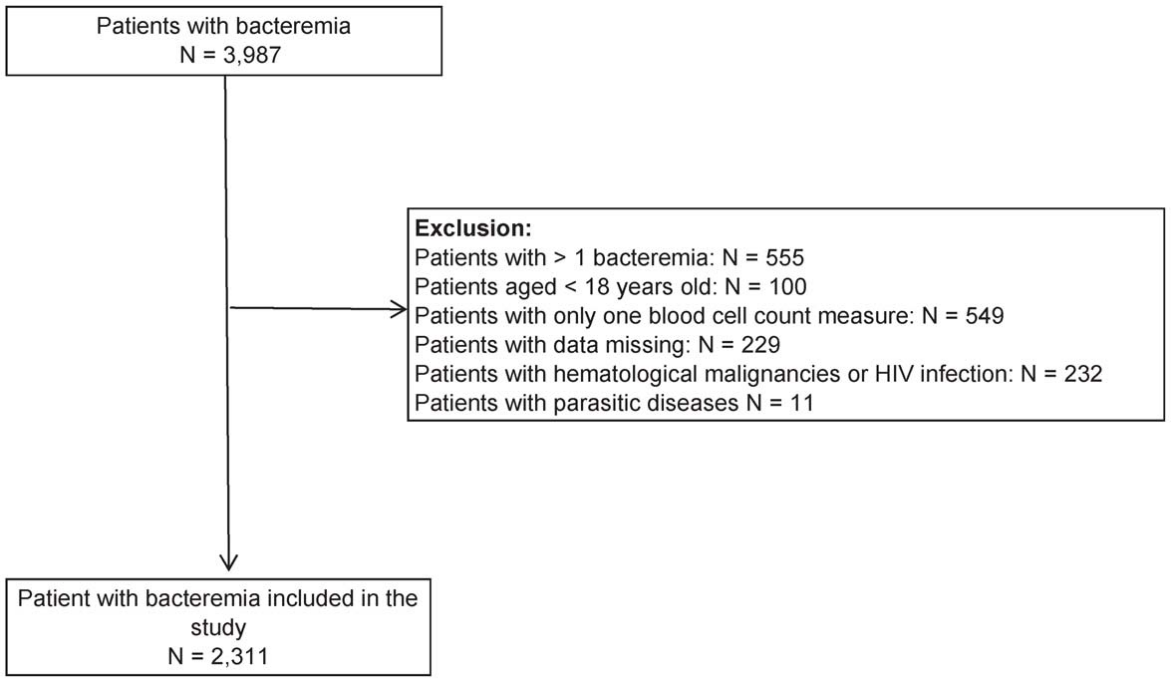
Cases analyzed, exclusion criteria and study population.

During the study period, there were 3,987 patients with a bacteremia episode. Once all exclusions were performed, 2,311 patients were included (Fig. 1).

Of the 2,311 patients, 255 (11.0%) died within 15 days. Of these, 111 (4.80% of the patients) died in the first 2 days and 131 (5.67%) in the first 3 days.

The mean length of hospital stay in patients with bacteremia was 10 days with IQR = 6–15 (11.5 days with IQR = 7–15 among survivors and survival of 3.0 days with IQR = 1–7 among non-survivors).

A total of 1,316 (56.95%) of the patients selected were men. Although there were a higher number of community-acquired episodes of bacteremia, mortality from healthcare-related bacteremia was 2.72 times higher (Table 1). Most of the patients (1,231) in our sample had a Charlson score of 2 or more. Moreover, mortality was higher in these patients than in those with a lower score (mortality rate: 13.3% with 95%CI = 11.4–15.2 versus 8.8% with 95%CI = 6.7–10.9). Corticosteroid treatment was administered in 191 patients, whose crude mortality rate was higher. Vasopressors were administered in 282 patients, who had a higher mortality rate than those not receiving these drugs (25.89% with 95%CI = 22.92–28.85 *vs.* 8.97 with 95%CI = 7.51–10.43). Admission to the intensive care unit and vasopressor exposure were similar, occurring in approximately 252 patients (10.9%). The mean age of survivors was 67.22 years compared with 71.51 years in non-survivors. The median age of non-survivors was higher than that of survivors (*p*<0.001).

**Table 1:** Patient characteristics in relation to mortality.

		Total	Death in the first 15 days	Chi-squared test
Variable	Categories	N	N (rate [%])	p-value
**Number of patients**		2,311	255 (11.0)	
**Age**	Mean (sd)	67.70 (16.26)	71.52 (14.11)	<0.001*
	Median	71.86	75.78	
	IQR	58.87−79.48	62.69–81.86	
**Sex**	Men	1,316	169 (12.8)	0.002
	Women	995	86 (8.6)	
**Place of acquisition**	Healthcare-related	840	155 (18.5)	<0.001
	Community-acquired	1,471	100 (6.8)	
**Charlson Index**	0	704	62 (8.8)	<0.001
	1	355	25 (7.0)	
	≥2	1,231	164 (13.3)	
	Unknown	21	4	
**Clinical Area**	Medical	1,588	140 (8.8)	<0.001
	Surgical	723	115 (15.9)	
**Source of bacteremia**	Urine	689	36 (5.2)	<0.001
	Surgery	98	11 (11.2)	
	Respiratory	268	46 (17.2)	
	Catheter	231	28 (12.1)	
	Abdominal non-surgical	391	42 (10.7)	
	Skin	115	11 (9.6)	
	Unknown	269	61 (22.7)	
	Others	250	20 (08.0)	
**Microorganisms**	*Escherichia coli*	739	53 (7.17)	<0.001
**isolated**	*Klebsiella spp*	198	23 (11.62)	
	*Pseudomonas aeruginosa*	103	29 (28.16)	
	Other Gram-negative microorganism	258	35 (13.57)	
	*Staphylococcus aureus*	187	25 (13.37)	
	*Streptococcus pneumoniae*	147	9 (6.12)	
	*Enterococcus spp*	74	12 (16.22)	
	Other Gram-positive microorganism	372	34 (9.14)	
	Anaerobics	94	10 (10.64)	
	Polymicrobial	90	10 (11.11)	
	Fungi	29	15 (51,72)	
	Unknown	20	0	
**Corticosteroid use**	No	2.120	213 (10.05)	<0.001
	Yes	191	42 (21.99)	
**Vasopressors use**	No	2.029	182 (8.97)	<0.001
	Yes	282	73 (25.89)	

sd: standard deviation.

IQR: Interquartile range.

* We used the Mann-Whitney U-test to compare the median age between survivors and non-survivors.

**Figure 2:**
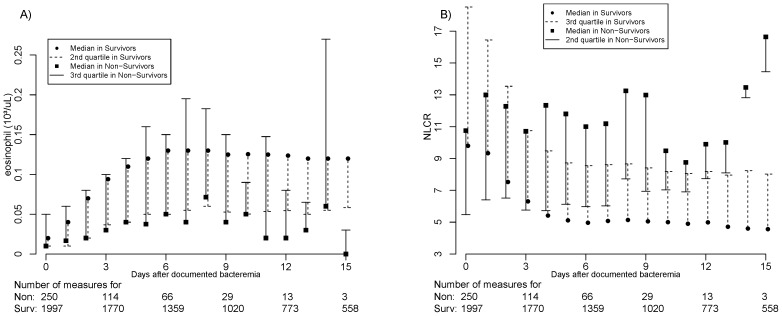
Median for eosinophil count and NLCR for survivors and non-survivors in each day. Legend: **2A**) The median eosinophil count for each day in survivors (circle) and non-survivors (square) in the first 15 days. The dashed line represents the second quartile of the eosinophil count on specific days in survivors. The continuous line represents the third quartile of the eosinophil count on specific days in non-survivors. The blood tests for each day and for survivors (Surv) and non-survivors (Non) are shown at the bottom of the figure. **2B**) The median of the NLCR count for specific days in survivors (circle) and non-survivors (square) in the first 15 days. The dashed line represents the third quartile of the NLCR count on specific days in survivors. The continuous line represents the second quartile of the NLCR count for specific days in non-survivors. The blood tests for each day and for survivors (Surv) and non-survivors (Non) are shown at the bottom of the figure.

Figure 2 shows the median eosinophil count (Fig. 2A) and the median NLCR (Fig. 2B) for survivors and non-survivors in each day of the first 15 days, as well as the number of blood tests performed in each group.

The trend in eosinophil count (Fig. 2A) showed that the median daily value was higher in survivors than in non-survivors (*p*<0.01 for each day except for the 14^th^ day, when *p* = 0.53). Between days 2 and 3, the median eosinophil count in survivors rapidly increased to the normal range (0.05–0.5·10^3^/uL). In more than half of non-survivors, the eosinophil count was always below the lower limit of normality.

The descriptive analysis of the NLCR (Fig. 2B) showed that the median value was lower in survivors after the day of documented bacteremia (*p*<0.01 for each day except the day that blood culture was performed, when *p* = 0.23). After day 3, the median value in survivors was always below the second quartile of the distribution of non-survivors.

**Figure 3:**
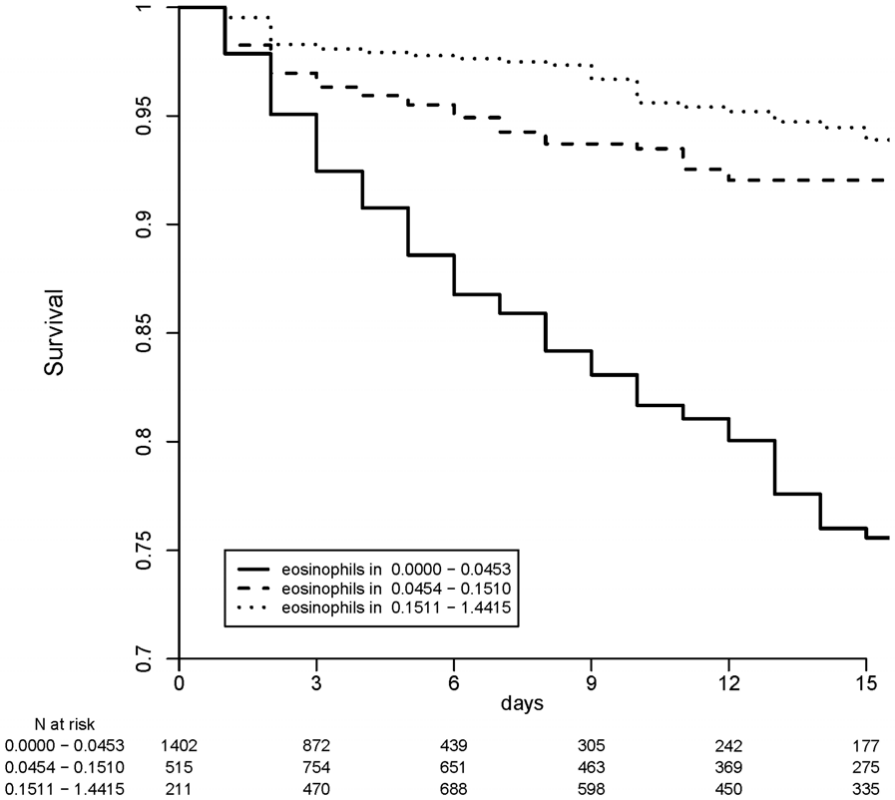
Survival curves according to eosinophil count. Legend: The continuous curve represents mortality in patients with an eosinophil count lower than 0.0454·10^3^/uL. The dashed line represents survival in patients with an eosinophil count from 0.0454–0.15·10^3^/uL. The dotted curve represents survival in patients with an eosinophil count higher than 0.15·10^3^/uL.

The Kaplan-Meier curve (Fig. 3) showed that mortality was higher in patients with eosinophil counts below 0.0454·10^3^/uL (*p*<0.001). In the first 3 days, mortality in the three groups did not differ but after the third day, mortality was higher in the group with counts that continued to be below 0.0454·10^3^/uL than in the remaining two groups. Likewise, mortality among patients with an eosinophil range between 0.0454 and 0.1510·10^3^/uL was higher than that in patients with a range between 0.1511 and 1.4415·10^3^/uL.

The unadjusted and adjusted estimations of the Cox model to evaluate the factors associated with survival at 15 days in bacteremia episodes are shown in Table 2. In both analyses, having an eosinophil count below 0.0454·10^3^/uL was the second most important risk factor for mortality. These patients had an HR of 4.20 higher than that of those with values above 0.15·10^3^/uL. In addition, patients with an NLCR >7 had a higher HR for mortality than those with an NLCR < = 7 (HR = 1.72). The analysis by different microorganisms indicated that only fungemia (main risk factor with HR = 4.26) or bacteremia caused by *Pseudomonas aeruginosa* (HR = 1.79) were significantly associated with higher mortality. Patients exposed to vasopressors had higher mortality (HR = 2.11). Although the univariate analysis showed a significant increase in mortality related to corticosteroid exposure (HR = 1.97), the adjusted analysis showed a protective effect of corticosteroids against mortality (HR = 0.55).

**Table 2:** Hazard ratios for the association between patient characteristics and mortality.

		Univariate	Multivariate
Variable	Categories	HR (95% CI)	HR (95% CI)
**Eosinophil count**	0.0000−0.0453·10^3^/uL	4.77 (3.15−7.23)	4,20 (2,66−6,62)
	0.0454−0.1510·10^3^/uL	1.55 (0.97−2.47)	1,53 (0,92−2,52)
	0.1511−1.4415·10^3^/uL	Ref	Ref
**NLCR**	NLCR ≤7	Ref	Ref
	NLCR >7	2.74 (2.01−3.74)	1,72 (1,24−2,39)
**Age**	Increase 1 year	1.02 (1.01−1.03)	1,02 (1,01−1,03)
**Sex**	Women	Ref	Ref
	Men	1.50 (1.16−1.95)	1,21 (0,90−1,64)
**Place of acquisition**	Community-acquired	Ref	Ref
	Healthcare-related	2.54 (1.98−3.27)	1,64 (1,16−2,32)
**Charlson Index**	0	Ref	Ref
	1	0.77 (0.48−1.22)	1,02 (0,60−1,72)
	≥2	1.42 (1.06−1.90)	1,27 (0,89−1,82)
**Clinical Area**	Medical	Ref	Ref
	Surgical	1.18 (0.91−1.53)	0,83 (0,60−1,16)
**Source of bacteremia**	Urine	Ref	Ref
	Surgery	1.79 (0.91−3.52)	0,85 (0,31−2,33)
	Respiratory	3.02 (1.95−4.67)	2,85 (1,65−4,91)
	Catheter	1.95 (1.19−3.20)	1,35 (0,71−2,58)
	Abdominal non-surgical	1.89 (1.21−2.94)	1,68 (0,99−2,85)
	Skin	1.54 (0.78−3.02)	2,11 (0,95−4,67)
	Unknown	4.10 (2.71−6.19)	2,91 (1,74−4,88)
	Others	1.28 (0.74−2.21)	1,56 (0,70−3,50)
**Microorganisms**	*Escherichia coli*	Ref	Ref
**isolated**	*Klebsiella spp*	1,50 (0,92−1,44)	1,16 (0,64−2,08)
	*Pseudomonas aeruginosa*	3,71 (2,36−5,85)	1,79 (1,03−3,10)
	Other Gram-negative	1,79 (1,17−2,75)	1,38 (0,84−2,28)
	*Staphylococcus aureus*	1,55 (0,96−2,49)	1,34 (0,75−2,36)
	*Streptococcus pneumoniae*	0,78 (0,38−1,58)	0,48 (0,20−1,15)
	*Enterococcus spp*	1,99 (1,06−3,72)	1,34 (0,65−2,73)
	Other Gram-positive	1,14 (0,74−1,76)	0,83 (0,46−1,49)
	Anaerobics	1,31 (0,67−2,58)	1,08 (0,51−2,28)
	Polymicrobial	1,34 (0,68−2,63)	0,90 (0,31−2,63)
	Fungi	8,05 (4,54−14,29)	4,26 (2,14−8,49)
**Corticosteroid use**	No	Ref	Ref
	Yes	1.97 (1.41−2.75)	0,55 (0,36−0,85)
**Vasopressor use**	No	Ref	Ref
	Yes	2.58 (1.97−3.39)	2,11 (1,51−2,94)

A stratified analysis separating patients exposed and not exposed to vasopressors was performed. The HRs for eosinophil count below 0.454·10^3^/uL were equal in the two models (HR = 4.28 [95%CI = 2.44–7.52] without vasopressors and HR = 4.83 [95%CI = 2.13–10.94] with vasopressors). The remaining variables had the same effect on both strata.

In the subanalysis to assess the prognostic value of the baseline eosinophil count in crude mortality at 3 days, an eosinophil count at blood extraction for culture was available in 2,605 patients. Of these, 112 (4.3%) died in the first 3 days. In the baseline blood test, the mean value of the leukocyte count was 10.6·10^3^/uL, eosinophil count was 0.02·10^3^/uL (IQR: 0.00–0.05·10^3^/uL) and the NLCR was 11.10 (IQR: 2.87–20.15). Analysis of crude mortality at 3 days according to the eosinophil count and the NLCR discriminated poorly between survivors and non-survivors at 3 days, since the area under the ROC curve was 0.61.

## Discussion

This study, conducted in a cohort of 2,311 patients with bacteremia, found that a below-normal eosinophil count (<0.05·10^3^/uL) was associated with a 4.77-fold increase in the HR of dying compared with a normal eosinophil count. The analysis adjusted by other variables showed that, independently of other factors, the second important risk factor for death was a persistently below-normal eosinophil count (HR = 4.20). A return to normal eosinophil count after the third day was found in survivors. A similar pattern was found in the NLCR. Although the median value of this ratio reached 11.10 during bacteremia episodes, a rapid decrease to below 7 was found to indicate good outcome.

Several studies [4]–[8] have suggested that eosinopenia can be a marker of bacterial infection in distinct types of patients. These studies include heterogeneous populations and have a small number of patients, representing a major limitation for their interpretation, which is reflected in their contradictory results. In the present study, in the initial determination, the mean eosinophil count was 0.02·10^3^ uL, a value that would support a presumptive association between eosinopenia and bacterial infection. However, this association could not be confirmed since it was not included in the study’s objective and design.

Abidi et al. [16] evaluated eosinopenia as an early marker of mortality in critically ill patients, a high percentage of whom had infection. In the multivariate analysis, eosinopenia was a predictor of mortality at 28 days with an HR of 1.8. Although drawn from a distinct type of patient, the findings of the present study support these results and, in addition, show their general applicability in patients throughout the hospital, on the one hand, and demonstrate their applicability to a specific infection (bacteremia), on the other.

Holland et al. [17] analyzed admission eosinophil count in 66 patients with exacerbation of chronic obstructive pulmonary disease and found that mortality was statistically significantly higher in patients with eosinopenia at baseline than in those with normal eosinophil values (17.4% versus 2.4%, respectively). These authors suggested that eosinophil count could be a useful marker of severity and prognosis independently of other, routinely used indicators. In patients with bacteremia, such as those included in the present study, the initial eosinophil count did not allow patient outcome to be predicted.

The NLCR was useful for diagnosis of bacteremia when the result was above 10 [2]. In the present study, an NLCR of below 7 was indicative of a favourable outcome.

This marker has also been used as an indicator of prognosis or mortality in distinct patient groups. In patients with lung cancer, NLCR was an independent marker of mortality [25]. In patients with colon cancer [26], high NLCR values were related to advanced stages, suggesting that this ratio could have prognostic value. In another group of patients with colon cancer [27], NLCR values above 9.3 were related to the risk of complications, although the authors of this study suggested that larger series were required to confirm this cut-off as an independent risk factor. In patients with liver cancer, high NLCR values were related to poor prognosis [28]. The NLCR was also used in a study of patients with acute coronary syndrome [29], in which high values were related to higher mortality on admission or in the first 6 months after discharge.

Exposure to vasopressors was found to be associated with increased mortality. In contrast, the association with corticosteroid exposure is more difficult to explain; in the univariate analysis, this factor was associated with increased mortality, but in the adjusted analysis it was related to lower mortality; these results probably reflect the fact that corticosteroid therapy was used in more severe patients, in whom it had a protective effect. The role of corticosteroids and vasopressor in the trend in eosinophil count is controversial. While Bass found no association between vasopressors, corticosteroids and eosinopenia [30], [31], Weller proposed that corticosteroids were associated with a reduction in eosinophil levels [32].

The analysis by different microorganisms is shown in Table 2, indicating that only fungemia or bacteremia caused by *Pseudomonas aeruginosa* were significantly associated with increased mortality, a finding that has been extensively described in the literature [33], [34].

The present study included only patients with bacteremia. Eosinopenia could be a non-specific marker of poor outcome or severity and may not be a specific marker of sepsis with poor outcome. This consideration is clinically relevant because if the specificity of eosinophil count were demonstrated, this marker could be used to guide the choice of complementary examinations or even empirical changes in antimicrobial therapy.

### Limitations

One of the limitations of this study is that the data are drawn from clinical practice and consequently, daily laboratory determinations are lacking in some patients. Another limitation is the number of patients lost to follow-up, both those who died early and those who improved rapidly and were discharged, since in both cases, the number of laboratory determinations was limited. However, the cohort of patients with bacteremia was large, lending strength to the associations found.

Since this study was retrospective, eosinophil count was not compared with other markers of outcome, such as procalcitonin or C-reactive protein. During the study period, there were a limited number of patients with more than one determination of these markers, which were not measured systematically over time for all patients. Experiences in patients with sepsis have shown that the sensitivity of procalcitonin is similar to that of eosinophil count, but with lower specificity [5].

Another limitation is the lack of a variable to identify the appropriateness of empirical antibiotic treatment. However, all patients were assessed by a bacteremia surveillance team, who reviewed and adjusted the treatments according Gram stain or antibiogram within 48 hours of bacteremia detection.

A further limitation was the lack of severity scores such as the Simplified Acute Physiology Score II (SAPS II) and Acute Physiology and Chronic Health Evaluation II (APACHE II). These scores are mainly used in the intensive care unit setting and, since the cohort of patients in the present study came from different areas of the hospital, comorbidities were assessed using the Charlson index.

### Conclusion

Our experience indicates that patients with bacteremia and persistent eosinopenia have a significantly increased risk of mortality. Moreover, those with an NLCR above 7 are also at higher risk of mortality. Therefore, eosinophil count and NLCR could be considered independent markers of outcome in patients with bacteremia. The use of some leukocyte counts as a marker of patient outcome is easy, rapid and inexpensive and consequently could be of use in daily clinical practice, especially in developing countries.
